# BMPs as new insulin sensitizers: enhanced glucose uptake in mature 3T3-L1 adipocytes via PPARγ and GLUT4 upregulation

**DOI:** 10.1038/s41598-017-17595-5

**Published:** 2017-12-08

**Authors:** Isabelle Schreiber, Gina Dörpholz, Claus-Eric Ott, Bjørt Kragesteen, Nancy Schanze, Cory Thomas Lee, Josef Köhrle, Stefan Mundlos, Karen Ruschke, Petra Knaus

**Affiliations:** 1 Institute of Chemistry and Biochemistry - Biochemistry, Berlin, Germany; 2 Berlin-Brandenburg School for Regenerative Therapies (BSRT), Berlin, Germany; 3 Institute for Human Genetics and Medical Genetics, Charité - Universitätsmedizin Berlin, Berlin, Germany; 4 Max Planck Institute for Molecular Genetics, Berlin, Germany; 5 Institute for Experimental Endocrinology, Charité-Universitätsmedizin Berlin, Berlin, Germany

## Abstract

Insulin-resistance is the main cause of type 2 diabetes. Here we describe the identification and characterization of BMP2 and BMP6 as new insulin-sensitizing growth factors in mature adipocytes. We show that BMP2 and BMP6 lead to enhanced insulin-mediated glucose uptake in both insulin-sensitive and -insensitive adipocytes. We exclude a direct effect of BMP2 or BMP6 on translocation of GLUT4 to the plasma membrane and demonstrate that these BMPs increase GLUT4 protein levels equipotent to Rosiglitazone. BMPs induce expression of PPARγ as the crucial mediator for the insulin-sensitizing effect. A comprehensive RNA-Seq analysis in mature adipocytes revealed regulation of both BMP/Smad and PPARγ target genes. The effects of BMP2 and BMP6 are not completely redundant and include regulation of genes involved in glucose and fatty acid metabolism and adipokine expression. Collectively, these findings suggest the BMP2 and BMP6 pathway(s) as promising new drug targets to treat insulin resistance.

## Introduction

As the number of people with diabetes has quadrupled since 1980, so has the need for new therapeutic interventions. The major aspect of type 2 diabetes is chronic hyperglycemia due to insulin resistance in muscle, fat, and liver. Insulin-stimulated glucose uptake is mediated by GLUT4, the most important glucose transporter in muscle and adipose tissue. GLUT4 is downregulated in diabetes patients and its expression levels are correlated with whole-body glucose homeostasis^1^. The antidiabetic drug Rosiglitazone, the strongest known PPARγ agonist, has been withdrawn from the European market since adverse drug reactions like severe cardiovascular events and increased bone fracture rates upon long-term use have been reported^2,3^. Hence, there is a strong need to identify new insulin-sensitizers and to unravel molecular processes involving PPARγ modulation in adult adipose tissue.

Bone/body morphogenetic proteins (BMPs) are secreted growth factors originally discovered by their ability to induce bone and cartilage formation^4^. There are more than 20 different members of BMPs known with distinct roles in organ development and homeostasis as shown by their individual gene knockout phenotypes. Beside their classical role in development, BMPs regulate several biological processes such as adipogenesis, iron metabolism in the liver, and differentiation and thermoregulatory activity of brown adipose tissue^5^. BMPs exert their function by binding to hetero-oligomeric complexes comprising of BMP type I (ACVR1A, BMPR1A, and BMPR1B) and type II (BMPR2, ACTR2A, and ACTR2B) receptors. Interestingly, also mechanical forces are integrated and contribute to the BMP signaling network^6^.

Genetic and evolutionary analyses revealed expression of the *BMP type II receptor* (*BMPR2*) gene in visceral and subcutaneous adipose tissue, which suggests a role for BMPR2 in the pathophysiology of human adiposity^7^. Moreover, expression levels and genetic variants of the *BMP type 1A receptor* (*BMPR1A*) gene correlate with obesity in humans^8^. Conditional ablation of *Bmpr1a* in aP2 (also known as FABP4) expressing tissues prevents age-related impairment of insulin sensitivity in normal and HFD fed mice. Further, the Adipoq-Bmpr1a-KO strain, in which mature adipocytes are more specifically targeted, recapitulates the observed phenotype in aP2-Bmpr1a-KO mice only partially, suggesting that BMP signaling is very versatile in mature adipocytes^9^. Interestingly, the distant TGF-β family member growth/differentiation factor 15 (GDF15) induces weight loss through decreased food intake in obese rodents and primates via its recently identified receptor GDNF Family Receptor Alpha Like (GFRAL) which is expressed in the area postrema of the brain^10–12^.

The most extensively studied member of the BMP ligand family is BMP2^13^. BMP2 and its closest relative BMP4 promote white adipogenesis in various precursor cell lines by induction of peroxisome proliferator-activated receptor (PPAR)-γ, whereas BMP7 exclusively promotes brown adipogenesis^14–17^. The white-to-brown transition of human preadipocytes is enhanced by BMP4 and BMP7^18^. In the context of brown adipogenesis, a potential cross-talk between insulin and BMP signaling has been proposed^19^. BMP4, BMP7, and BMP9 are expressed in adipose tissue and act as adipokines regulating glucose homeostasis in several other tissues^20–23^. Being the first BMP detected in the blood stream of healthy individuals, BMP6 is best known for its role in iron metabolism^24^. However, BMP6 has been proposed to induce brown fat differentiation of skeletal muscle precursor cells^25^. Recently, the expression of BMP2, especially in visceral adipose tissue, was found to positively correlate with BMI and diabetic status in the Leipzig Cohort^26^. In mature adipocytes, BMP signaling has never been investigated systematically. Moreover, BMP2 and BMP7 are clinically used to promote bone healing in cases of bone fracture nonunions and in spine surgery. Taken together, there is an emerging need to understand the precise role of BMP signaling in adult tissues, particularly in the context of glucose metabolism.

Here we show that both BMP2 and BMP6 act on mature adipocytes as insulin-sensitizers. BMP signaling leads to transcriptional upregulation of the adipocyte master regulator PPARγ, which in turn upregulates GLUT4 and several other genes involved in lipid metabolism as revealed by whole transcriptome analysis.

## Results

BMP2 and BMP4 are well known for their function in white adipogenesis and BMP2 was proposed to play an important role in energy storage partitioning and obesity^26^, while BMP7 is involved in brown adipogenesis^14^. The role of BMP6, structurally most similar to BMP7, in adipocytes has never been addressed. In addition, the physiological relevance and molecular mode of action of BMPs in adult adipose tissue remain largely elusive. To uncover the role of BMP signaling in mature i.e. terminally differentiated adipocytes and adult glucose metabolism, we conducted a comparative study addressing the impact of BMP2 and BMP6 on signal transduction, gene expression and metabolism.

For this purpose, we used 3T3-L1 cells which were terminally differentiated, stimulated with BMP2 or BMP6 and subjected to various molecular and physiological analyses. To assess the efficiency of 3T3-L1 differentiation, cells were stained for adipocyte-typical lipid droplets with the lipophilic fluorochrome BODIPY 493/503 and analyzed via flow cytometry (Supplementary Fig. S1a) and microscopy (Supplementary Fig. S1b). Eight days after induction, differentiation efficiency was >90%.

### BMP2 and BMP6 induce P-Smad, P-p38 signaling and upregulate PPARγ

**Figure 1:**
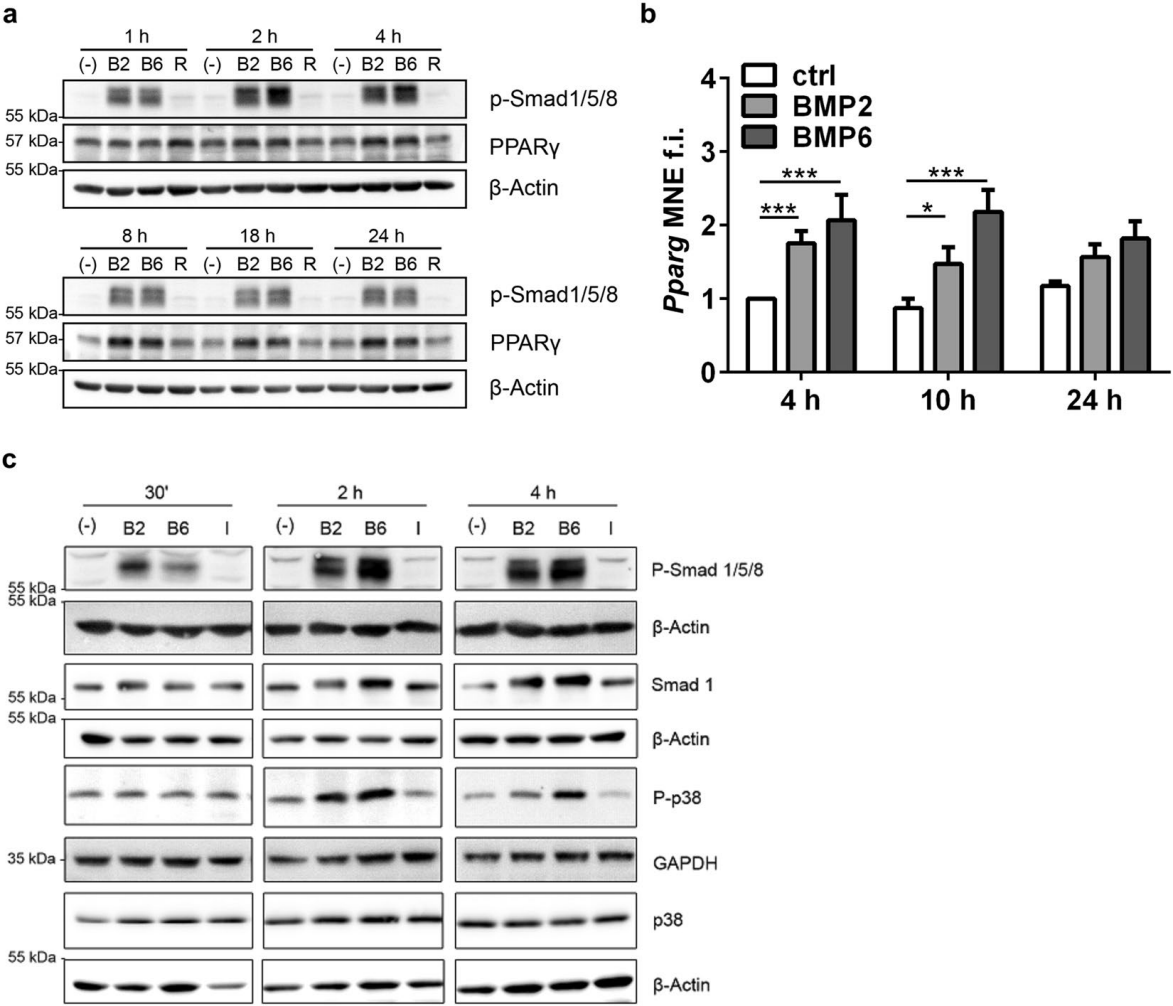
BMP2 and BMP6 upregulate PPARγ, a crucial mediator of GLUT4 expression. (**a**) Adipocytes were stimulated with PBS (−), BMP2 (B2), BMP6 (B6) or Rosiglitazone (R) for 1 h to 24 h and lysates were analyzed using indicated antibodies via immunoblotting. (**b**) 3T3-L1 adipocytes were stimulated with PBS, BMP2 or BMP6 and mRNA expression was analyzed via qRT-PCR at the time points indicated. Data are means + SEM of 4 independent experiments with triplicate measurements each. Statistical significance was calculated using the Kruskal-Wallis test. (**c**) Immunoblot analysis of BMP signaling in mature 3T3-L1 adipocytes. Mature adipocytes (d8) were stimulated with PBS (−), 10 nM BMP2 (B2), 10 nM BMP6 (B6), 10 µM Rosiglitazone (R) or 100 nM insulin (I) for indicated time points. The panels of the immunoblots are derived from separate membranes and are not cropped.

To characterize BMP signaling in adult adipocytes, we performed time-resolved stimulation experiments assessing protein phosphorylation via immunoblotting. BMP2 as well as BMP6 treatment led to Smad1/5/8 phosphorylation after one hour, which persisted at least 24 h (Fig. 1a). Of note, Smad1/5/8 phosphorylation by BMP6 was stronger after 2 h and 4 h of stimulation compared to BMP2. Moreover, BMP2 and BMP6 induced phosphorylation of the MAP kinase p38 (Fig. 1c). As shown previously for BMP2-mediated white adipocyte differentiation in C3H10T1/2 cells, both Smad1 and p38 signaling are responsible for the upregulation of the master regulator PPARγ and its transcriptional activity^27^. Beside its crucial role in adipogenesis, PPARγ is also thought to play an important role in the maintenance of mature adipocyte function controlling the expression of metabolic key enzymes and glucose transporters^28^. Remarkably, PPARγ protein was most upregulated after 8 h of BMP stimulation (Fig. 1a and Supplementary Fig. S1f. *Pparγ* mRNA expression was significantly induced in mature adipocytes after 4 and 10 h by both BMP2 and BMP6 (Fig. 1b). Thus, BMP2 or BMP6 signaling leads to upregulation of the transcription factor and nuclear receptor PPARγ in terminally differentiated adipocytes.

### Effects of BMP2 and BMP6 on mature adipocytes are not redundant

**Figure 2:**
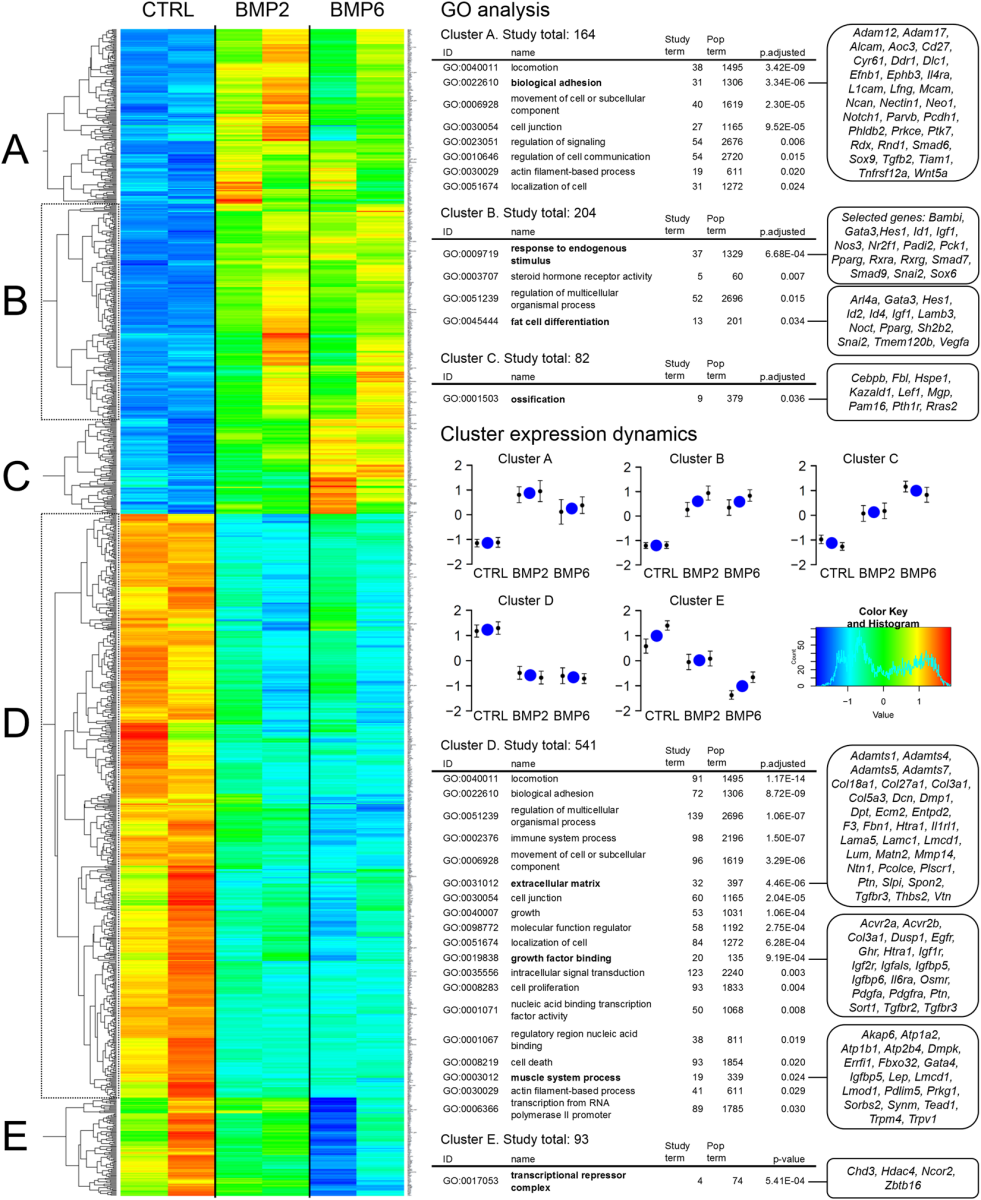
BMP stimulation of mature adipocytes alters biological and cellular processes related to growth factor signaling, fat cell differentiation and transcriptional regulation. Cluster analysis for RNAseq data of 2 biological replicates. The union of BMP2 and/or BMP6 regulated genes with more than 1.5 fold change and an adjusted p-value < 0.01 (1084 genes) were selected for GO term analysis. Hierarchical clustering resulted in five clusters (**A**–**E**) on which GO analyses with MGSA were executed using Ontologizer^60^. More unspecific GO terms with a population size >2500 were excluded. Prominent GO terms are highlighted and corresponding genes are shown on the right-hand side.

To further investigate the transcriptional response of mature adipocytes to BMP2 and BMP6 treatment, we performed gene expression profiling. Upon 10 h of BMP2 or BMP6 treatment 800 genes and 965 genes respectively (Supplementary Fig. S1c and d) were significantly differentially expressed (p < 0.01) with an overlap of 648 genes. Model-based gene set analysis revealed GO terms such as ‘*fat cell differentiation’*, ‘*response to cytokine*’, and ‘*intracellular signal transduction*’ (Table 1). Hierarchical clustering resulted in five major clusters (Fig. 2). Cluster A comprises genes that display slightly higher upregulation after BMP2 treatment. Here, 54 of 164 genes are annotated to ‘*regulation of signaling*’ and 31 genes to ‘*biological adhesion*’. Cluster B refers to genes, which are similarly upregulated by BMP2 and BMP6. GO analyses revealed that 13 of 204 genes are annotated to ‘*fat cell differentiation*’ and 37 genes to ‘*response to endogenous stimulus*’. This cluster includes *Pparg, Rxrα*, and *Smad9* (also known as *Smad8*) and well-known BMP targets like *Id1-4*. Genes in cluster C are slightly more responsive to BMP6. Nine out of 82 genes in this cluster are annotated to ‘*ossification*’. Cluster D contains almost half of all differentially expressed genes (541 genes). These genes are downregulated by BMP2 and BMP6 and annotated to ‘*extracellular matrix’, ‘growth factor binding*’, and ‘*muscle system process*’. Genes in cluster E are slightly more downregulated upon BMP6 treatment (93 genes). Downregulated genes include receptors like *Acvr2a, Acvr2b, Tgfbr2, Tgfbr3*, and ‘*transcriptional repressors*’ such as *Hdac4* and *Zbtb16*. Both BMP ligands have similar effects on a great number of genes, but subsets of genes are regulated exclusively by BMP2 (152 genes) or BMP6 (317 genes).

**Table 1:** Overview of biological processes annotated to differentially expressed genes upon BMP stimulation.

ID	Name	Marginal	Count
GO:0045444	fat cell differentiation	0.869	45/201
GO:0034097	response to cytokine	0.781	69/541
GO:0001501	skeletal system development	0.777	72/469
GO:0035556	intracellular signal transduction	0.729	231/2240
GO:0042383	sarcolemma	0.523	23/143
GO:0030036	actin cytoskeleton organization	0.504	71/543

Mature adipocytes (d8) were stimulated with PBS, 10 nM BMP2 or BMP6 for 10 h. Isolated total RNA was subjected to library preparation and deep sequencing (Illumina) to assess total gene expression profiles, n = 2 per condition. The union of 1084 differentially expressed genes (DEseq. 2) were analyzed as the study set in comparison to 24,211 mapped murine genes. The column ‘marginal’ indicates the marginal probability of a term being in the ‘active’ state, and the column ‘count’ shows the counts of genes in the study (x) and population (y) sets as x/y. Analysis was performed 5 times and most frequent terms were ranked according to their marginal value.

### PPARγ-target identification and validation

**Figure 3:**
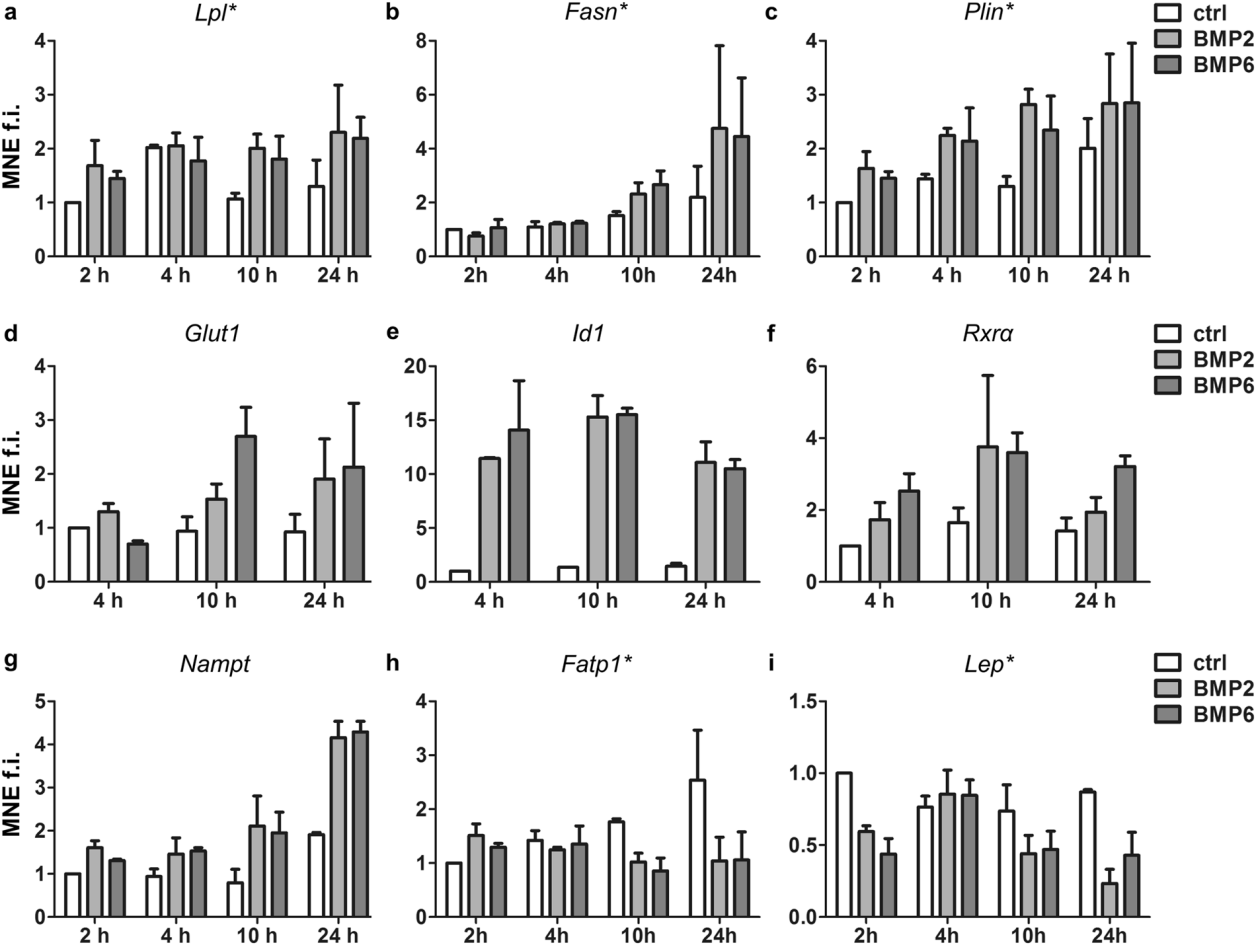
BMP2 and BMP6 stimulation regulate mRNA levels of metabolic enzymes/transporters and visfatin. Validation of genes responding to BMP stimulation by qRT-PCR. Adipocytes were treated as outlined in Fig. 2. Data are presented as means + SEM of two independent experiments different from samples used in the RNAseq experiment, n = 2 for a-c,e-i; n = 3 for d. *Lpl* (**a**) and *Fasn* (**b**) were selected from Cluster A. *Plin1* (**c**), *Id1* (**e**) and *Rxra* (**f**) and *Nampt* (**g**) coding for Pre-B-cell colony-enhancing factor 1 (PBEF1) or visfatin were selected from Cluster B. *Glut-1* (**d**) was not found to be significantly regulated in the RNA-Seq approach, but selected for validation as it represents the second glucose transporter present in adipocytes. *Fatp1* (**h**) and *Lep* (**i**) were selected from Cluster D. Asteriks denote PPARγ target genes.

Motivated by these results, we compared our data to the putative PPARγ target genes identified by Nakachi *et al*.^29^. Indeed, we found 20 from 61 validated PPARγ targets and confirmed BMP-mediated differential expression for further 24 targets that contain a PPARγ response element (PPRE) in or in close proximity to their promoter region (Supplementary Table 1). Finally, we validated our findings in independent experiments. The PPARγ regulated genes *Plin1* (Cluster B) and *Lpl* (Cluster A) were upregulated at 2 h, while *Fasn* (Cluster A) expression increased at 10 h (Fig. 3a–c). Gene expression of *Glut1* (also known as *Slc2a1*), coding for glucose transporter 1 that is considered to reside constitutively at the plasma membrane of numerous cell types^30^, was not significantly regulated in our RNA-Seq data, but moderately upregulated by BMP2 and BMP6 as assessed by qPCR (Fig. 3d). In line with the RNA-seq data, the classical BMP-Smad-target *Id1* and the PPARγ coreceptor *Rxrα* (Cluster B) were upregulated in BMP-stimulated samples at all time points analyzed (Figs 3e and f, Supplementary Fig. S1e). Moreover, *Nampt*, coding for the adipokine Visfatin was strongly upregulated after 24 h of BMP treatment (Fig. 3g). Contrary, long-chain fatty acid (LCFA) transporter Fatp1 (*Scl27a1*) and Leptin (*Lep* or *Ob*) found in Cluster D of the RNA-Seq results were downregulated by both BMP ligands after 10 h and 24 h of stimulation (Fig. 3h and i). Downregulation of the receptors *Acvr2a, Acvr2b* as well as *Igf1r* and *Igf2r* after 10 h of BMP stimulation was validated by qRT-PCR (Supplementary Fig. S1g). Moreover, expression of the histone deacetylase 4 (*Hdac4*), a known negative regulator of GLUT4 (cluster E)^31–33^ was validated to be diminished after BMP-stimulation (Supplementary Fig. S3f). Taken together, we confirmed the BMP-specific modulation of genes from several Clusters of our RNA-Seq approach. In general, BMP stimulation of mature adipocytes not only upregulates PPARγ, but also modulates the expression of several genes involved in glucose and fatty acid metabolism/transportation and the adipokines Leptin and Visfatin.

### BMP2 and BMP6 augment total GLUT4 levels

**Figure 4:**
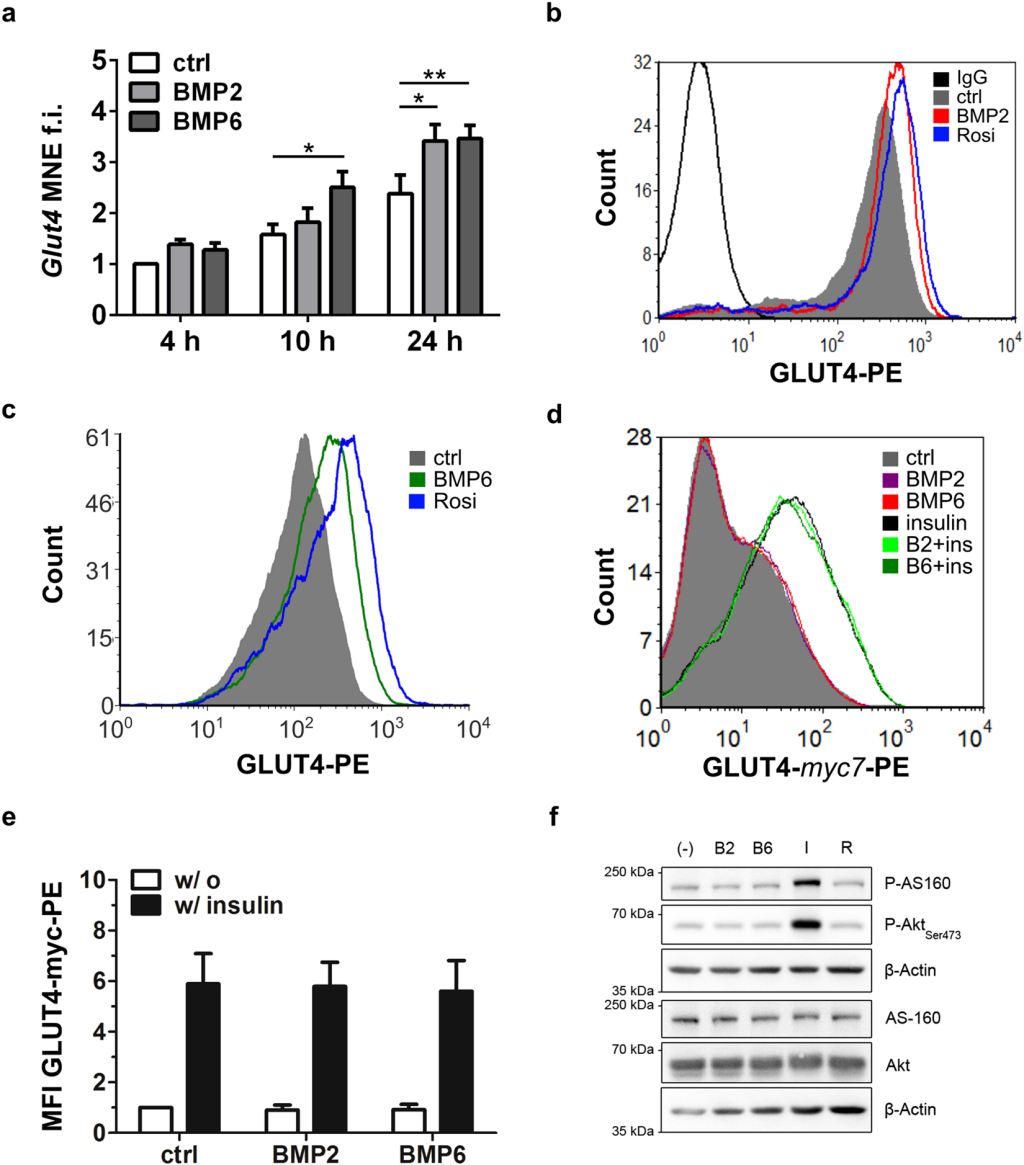
BMP signaling in adipocytes upregulates total levels of GLUT4, but does not interfere with insulin-stimulated GLUT4 translocation to the plasma membrane. (**a**) 3T3-L1 adipocytes were stimulated with PBS, BMP2 or BMP6 and analyzed after 4 h, 10 h and 24 h of stimulation for *Glut4* expression via qRT-PCR. Data are means + SEM of 4 independent experiments with triplicate measurements each. Statistical significance was calculated using 1-way ANOVA and Bonferroni post-hoc test. (**b**,**c**) Protein level of GLUT4 as determined via intracellular immunostaining and flow cytometric analysis gated on mature adipocytes. Representative histograms. (**d**,**e**) GLUT4myc7-GFP overexpressing 3T3-L1 adipocytes^36^ were used to monitor GLUT4 translocation to the cell surface. Cells were stimulated with control or insulin in combination with BMP2 or BMP6 for 30 minutes as indicated. **(e**) Data are means of multiple flow cytometric measurements (MFI) as fold induction of control + SEM, n = 3. (**f**) Analysis of AS160 phosphorylation as a critical step upstream of GLUT4-storage vesicle (GSV) fusion with the plasma membrane. Mature 3T3-L1 cells were stimulated with PBS (−), BMP2 (B2), BMP6 (B6), Insulin (I) or Rosiglitazone (R) for 30 minutes. Lysates were analyzed by immunoblotting with indicated antibodies, β-Actin served as a loading control. MFI = Mean Fluorescence Intensity, MNE = Mean Normalized Expression.

Both during adipocyte differentiation and after thiazolidinedione treatment in adult tissues^34^, GLUT4 expression is enhanced via PPARγ. Thus, we investigated changes of *Glut4* expression on the transcriptional level. As shown in Fig. 4a, *Glut4* mRNA expression was significantly induced 10 and 24 h upon BMP2 or BMP6 treatment. This effect was more pronounced for BMP6 and strongest upregulation was observed after 24 h. To assess the upregulation of GLUT4 protein, we performed intracellular immunostainings. Mature adipocytes were analyzed by flow cytometry with appropriate gating for size and granularity (Fig. 4b and c). Thereby, it was shown that BMP2 treatment led to a similar increase of GLUT4 protein as Rosiglitazone, while GLUT4 upregulation by BMP6 was slightly lower (Fig. 4b and c).

### BMP2 and BMP6 exhibit no direct effect on insulin signaling

Recently, BMP7 was found to enhance insulin-stimulated glucose uptake in insulin sensitive tissues by increasing PI3K/Akt signaling and subsequently GLUT4 translocation^35^. Therefore, we investigated the effect of BMP2 and BMP6 on the cell surface exposure of GLUT4 derived from GLUT4 storage vesicles (GSVs). 3T3-L1 cells stably expressing GLUT4myc7-GFP (G7-adipocytes) allow a discrimination between total and surface located GLUT4^36^. This is achieved via a C-terminal GFP-tag, which remains intracellular and 7 myc-tags in the first extracellular loop of GLUT4, which are available for antibody staining upon GLUT4 translocation to the surface. Differentiated G7-adipocytes were stimulated with BMP2, BMP6, insulin or combinations thereof for various periods and the abundance of surface GLUT4 was analyzed via flow cytometry (Fig. 4d). Insulin stimulation led to a clear shift in the GLUT4-myc7-PE signal, but no BMP effect on control (basal) or insulin-stimulated GLUT4 translocation was measured. Quantification of Mean Fluorescence Intensities (MFI) from multiple experiments confirmed that insulin-mediated GLUT4 translocation is not altered by treatment with either BMP2 or BMP6 (Fig. 4e). These findings suggest that there is no direct effect of BMP-signaling on GLUT4 translocation to the plasma membrane of differentiated adipocytes. Taken together, BMP2 and BMP6 did not directly affect the translocation of GLUT4 to the plasma membrane.

The BMP and insulin pathways share multiple signaling components and were proposed to cross talk during brown adipogenesis^19^. Therefore, we investigated putative BMP effects on the phosphorylation status of Akt substrate of 160 kDa (AS160, also known as TBC1D4), which is the most proximal signaling step before GSV fusion to the plasma membrane. We stimulated differentiated 3T3-L1 adipocytes with BMP2, BMP6, insulin or Rosiglitazone for different periods and analyzed the phosphorylation status of insulin signaling components. Both AS160 and Akt (Ser473) were phosphorylated by insulin but not by BMP stimulation (Fig. 4f, Supplementary Fig. S2a). Moreover, we observed an insulin-sensitizing effect 4 hours after BMP2 or BMP6 stimulation (Supplementary Fig. S2b and c). Taken together, BMP2 and BMP6 did not affect the translocation of GLUT to the plasma membrane or interfere with insulin signaling but augmented total GLUT4 levels within the cell. Therefore, we conclude that BMP signaling modulates glucose transport by a rather indirect mechanism most likely via *de novo* protein synthesis.

### BMP2 or BMP6 treatment enhances insulin-mediated glucose uptake in 3T3-L1 adipocytes

**Figure 5:**
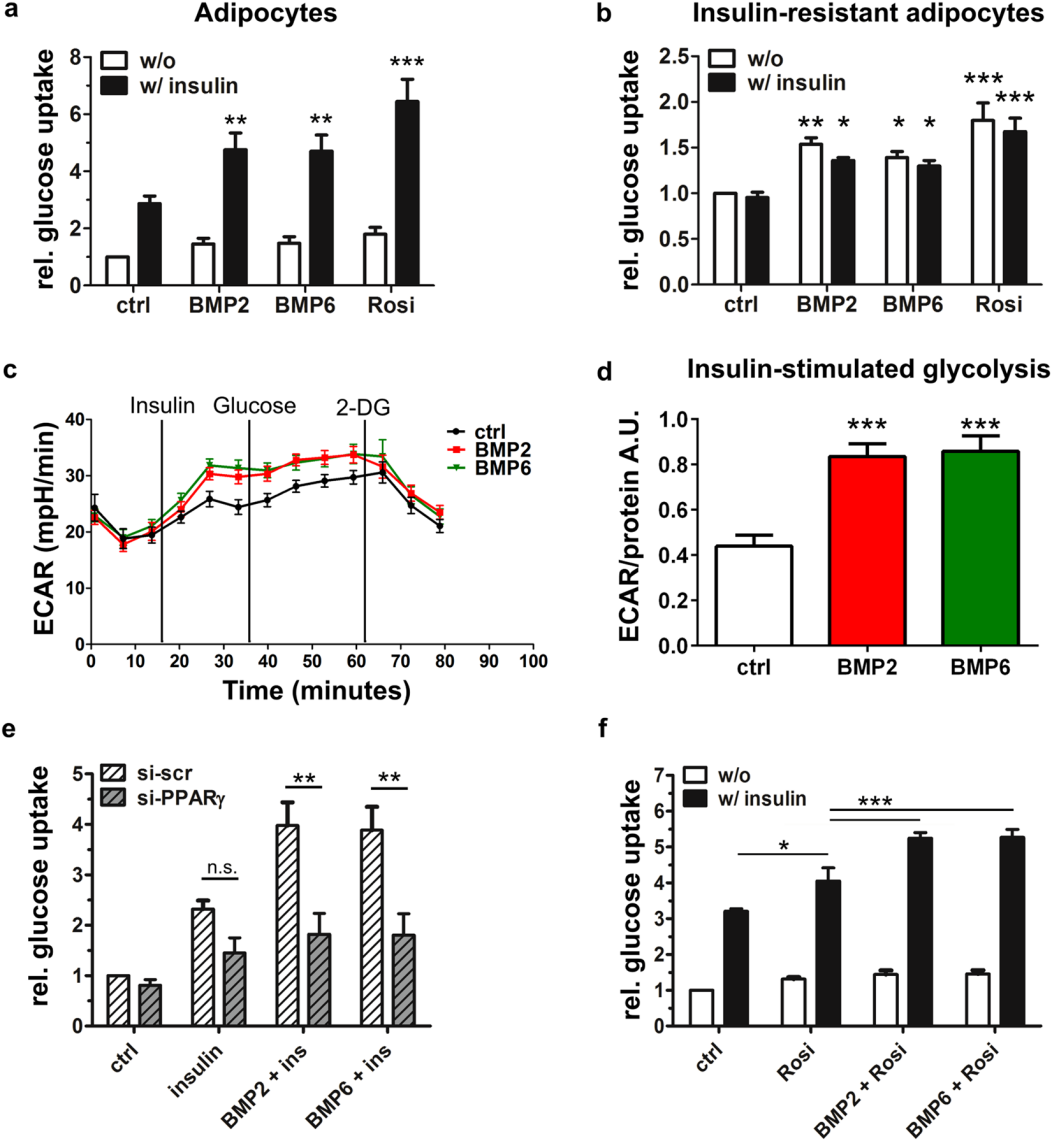
Activation of the BMP pathway leads to enhanced insulin-stimulated glucose uptake and glycolysis in mature adipocytes. (**a**,**b**) Mature adipocytes (d8) were pretreated with PBS, BMP2, BMP6 or Rosiglitazone for 18 h as indicated. 2-deoxy-D[^3^H] glucose uptake of adipocytes was measured after stimulation with PBS (white bars) or 100 nM insulin (black bars) for 30 minutes. Rosiglitazone was used as a positive control. (**a**) To test for statistical significance ≥seven independent experiments with three replicates each were analyzed using the 2-way ANOVA and Bonferroni post-hoc test. (**b**) *In vitro*-generated insulin-resistant 3T3-L1 adipocytes were stimulated as described in (**a**). Means + SEM of 3 independent experiments with triplicate measurements each are presented as fold induction of basal glucose uptake normalized to total protein content. Statistical significance was calculated three using the 2-way ANOVA and Bonferroni post-hoc test. (**c**) ECAR of 3T3-L1 adipocytes (d8) was recorded over time with a Seahorse XFe96 extracellular flux analyzer. Cells were kept in assay medium (5.5 mM glucose) and injected with 100 nM insulin, 10 mM glucose or 100 mM 2-DG at the time points indicated. 2-DG was injected to inhibit glycolysis. Representative measurement, data are means + SEM of 6 independently stimulated wells. (**d**) Insulin-stimulated glycolysis, which was defined as the difference between measurement 3 (18 minutes) and measurement 6 (36 minutes), normalized to total protein quantity, was calculated. Data are means + SEM of at least 3 independent experiments with six to twelve replicates each. Statistical significance was calculated using the Kruskal-Wallis test. (**e**) 3T3-L1 adipocytes were transfected with scrambled siRNA (si-scr) or siRNA targeting PPARγ (si-PPARγ) and incubated for 24 h before stimulation with BMPs, insulin or Rosiglitazone and subsequent measurements of 2-deoxy-D[^3^H] glucose uptake ± insulin (30 min). Data are means + SEM of at least 3 independent experiments with triplicate measurements each. Statistical significance was calculated using 2-way ANOVA and Bonferroni post-hoc test. (**f**) Simultaneous stimulation of mature adipocytes with Rosiglitazone and BMPs further enhanced the effect of Rosiglitazone on insulin-stimulated glucose uptake. Data are means + SEM of at least 3 independent experiments with triplicate measurements each. Statistical significance was calculated using 2-way ANOVA and Bonferroni post-hoc test.

To address the physiological role of BMP-mediated upregulation of PPARγ and GLUT4, insulin-stimulated glucose uptake, as one of the major functions of adipocytes, was analyzed. Mature adipocytes were stimulated with PBS (negative control), BMP2, BMP6 or the known insulin-sensitizer Rosiglitazone (positive control) for 18 h. Glucose uptake was measured after treatment with insulin or PBS for 30 minutes. Similar to Rosiglitazone, BMP2 and BMP6 pretreatment led to significantly higher insulin-mediated glucose uptake rates, as compared to control (1.5 fold, Fig. 5a). Remarkably, this effect was specific to BMP pathway activation, as pretreatment with the BMP type I receptor inhibitor LDN193189 abolished this effect (Supplementary Fig. S3d).TGF-β_1_, another member of the ligand family, had no impact on glucose uptake emphasizing a BMP-specific effect (Supplementary Fig. S2d and e).

Via chronic insulin stimulation we generated insulin-resistant 3T3-L1 adipocytes, which serve as an established model for adipose tissue insulin-resistance in type 2 diabetes^37,38^. Pretreatment with BMP2, BMP6, or Rosiglitazone significantly increased glucose uptake in these cells independent of an insulin pulse (Fig. 5b). In order to validate these findings and to gain more insight into the cellular energy metabolism, we next investigated the metabolic capacity using a Seahorse XFe96 Extracellular Flux Analyzer. The Seahorse technique allows the characterisation of metabolic changes in real-time by simultaneous measurement of the extracellular acidification rate (ECAR), indicative of glycolysis and the oxygen consumption rate (OCR), indicative for mitochondrial respiration^39^. After three baseline measurements, insulin was injected to stimulate glucose uptake. BMP pretreatment led to an increase in ECAR (i.e. more rapid decrease in pH over time) compared to control and a respective decrease in OCR (Supplementary Fig. S2f and g). By injecting supplementary glucose at minute 36, a slight increase in ECAR persisted in all conditions. The injection of 2-Deoxyglucose (2-DG), a competitive inhibitor of glycolysis, caused a rapid drop in ECARs for all samples thereby verifying that the measured ECAR corresponded to changes in the glycolytic rate (Fig. 5c). Multiple independent extracellular flux experiments revealed that insulin-stimulated glycolysis was significantly enhanced in BMP2 or BMP6 treated mature adipocytes (Fig. 5d). Data obtained from tritium-labeled glucose uptake and metabolic measurements provide clear evidence, that both BMP2 and BMP6 promote glucose uptake and utilization via glycolysis in mature adipocytes. Finally, we tested, whether the observed upregulation of PPARγ is crucial for the BMP effect on glucose uptake. Mature 3T3-L1 adipocytes were electroporated with siRNA targeting *Ppparγ* at day 6 of differentiation, resulting in a knockdown efficiency of 60% at day 7 (Supplementary Fig. S3a and b). Knockdown of *Ppparγ* reduced *Glut4* mRNA levels particularly in BMP stimulated cells, whereas other genes like the BMP/Smad target *Id1* remained unaffected (Supplementary Fig. S3e). In contrast to si-scr transfected control cells, the enhancement of insulin sensitivity upon BMP stimulation was nearly completely abolished in PPARγ depleted cells (Fig. 5e). Notably, the slight reduction of insulin-stimulated glucose uptake by siPPARγ (~20% reduction) was not statistically significant when compared to si-scr. Rosiglitazone as the strongest known agonist of PPARγ enhances its transcriptional activity on target genes like *Glut4* ^40^. Compared to the insulin-sensitizing effect of Rosiglitazone alone, the combination of Rosiglitazone and BMP stimulation enhanced the insulin-stimulated glucose uptake significantly (Fig. 5f).

Taken together, our data suggest that the BMP-dependent upregulation of the nuclear receptor PPARγ is crucial for the insulin-sensitizing BMP effect. The BMP effect on glucose uptake can be further enhanced by the presence of Rosiglitazone.

## Discussion

The identification and development of pharmacological insulin-sensitizers has led to promising ways to reduce insulin-resistance. However, especially the use of Rosiglitazone is under debate because of its side effects including cardiovascular complications and increased bone resorption. Since diabetes increases fracture risk with concomitant impaired healing capacities^41^, there is a strong need for further treatment strategies.

The expression of BMP2 in AT correlates with obesity parameters and diabetes status in patients, but the causality remains to be determined^26^. BMP6 is expressed in adult AT and also circulates in the blood^23,24^. However, its role in adult glucose/lipid metabolism has never been addressed. BMP2 promotes white adipogenesis in different precursor cells via Smad1/5/8 and Schnurri-2 (Shn-2)^18,27^. In contrast, BMP7, structurally similar to BMP6 but providing unique receptor binding properties, was shown to exclusively promote browning of human white adipocyte precursors and brown adipocyte differentiation^18,42^. BMP4, which is closely related to BMP2 was recently found to be upregulated in the serum of two diabetic mouse models and was proposed to contribute to insulin resistance by inhibiting IRS-1 activation and insulin signaling^35^. Furthermore, in a recently published gene therapy study, increased circulating BMP4 levels were shown to protect adult HFD-fed mice from obesity and improved their metabolic profile possibly due to increased browning of subcutaneous WAT^17^.

Here, we show that both BMP2 and BMP6 augment insulin-sensitivity in mature adipocytes. The effect on insulin-sensitive, as well as insulin-resistant adipocytes, is specific to BMPs since TGF-β1 stimulation had no influence on basal or insulin-stimulated glucose uptake. BMP2 predominantly binds BMPR1A whereas BMP6 shows the highest affinity towards ACVR1A^43^, but both BMPs use BMPR2, ActR2a or ActR2b as their type II receptors. All these receptors are expressed in 3T3-L1 adipocytes as well as in mature adipocytes (Supplementary Fig. S3c). Surprisingly, BMP6 treatment resulted in neither upregulation of brightening markers such as UCP-1 in 3T3-L1 cells nor increased OCR (Supplementary Fig. S2f). Although both BMP ligands have been reported to exert very different effects on adipogenesis, we demonstrate here that they have comparable effects on glucose uptake.

Glycolysis is the key process for energy storage in adipocytes and its modulation important to restore systemic glucose homeostasis^44^. In line with the glucose uptake data, the slope of the ECAR as a measure for cellular glycolysis was 2-fold increased in BMP-treated samples. Interestingly, many genes coding for glycolysis and lipogenesis enzymes like *Hk2*, *Fasn* and *Scd1* are upregulated upon BMP stimulation as seen in our RNA-seq analyses. Recent studies indicate that higher GLUT4 levels in mice correlate with *de novo* lipogenesis bearing antidiabetic and anti-inflammatory effects on adipose tissue^45^. This is in accordance with our observations.

Chattopadhyay *et al*. recently described an insulin-sensitizing function for BMP7 in insulin-sensitive tissues *in vivo* and attributed this to enhanced PI3K/Akt signaling and subsequent GLUT4 translocation to the cell surface^35^. However, using the GLUT4myc7-GFP cell line to monitor GLUT4 translocation to the cell surface of intact cells, we show that BMP2/6 signaling does not directly influence GLUT4 storage vesicle translocation. Both results on translocation of GLUT4 to the cell surface and on AS160 phosphorylation by Akt, clearly demonstrate no direct effect of BMP2 and BMP6 on this branch of the insulin signaling pathway. Akt signaling, which is activated by BMP signaling in many cell types represents a pro-survival signal. Moreover, the presence of high glucose was found to activate basal TGF-β/Smad signaling and the PI3K/Akt/mTOR pathway in MEFs^46^. We investigated a putative crosstalk of both pathways on this level, but neither Akt nor AS160 phosphorylation was observed after BMP stimulation. This is in line with our observations that BMP signaling alone as well as short-term stimulations (Supplementary Fig. S2b) of BMP2/6 together with insulin did not increase glucose uptake rates, clearly points towards changes on the transcriptional level.

Indeed, we observed an upregulation of *Glut4* mRNA upon BMP stimulation especially after 24 h. Earlier studies have shown that PPARγ ligands such as Rosiglitazone induce *Glut4* transcription during adipogenesis^47^. In line with this, *Hdac4* a negative regulator of *Glut4* expression was found to be downregulated, thus enabling upregulation of the glucose transporter. Here we demonstrate that BMP2 and BMP6 increased GLUT4 protein levels comparable to Rosiglitazone. This is of particular interest with regard to studies demonstrating that physical training is the strongest stimulus to enhance GLUT4 expression in skeletal muscle^48^. As the BMP/Smad pathway was shown in several cell types to be activated upon mechanical/physical stimuli^6^, this link might indicate GLUT4 as a potential downstream effector of both biochemical and biomechanical signaling in muscle but also other tissues such as AT. Moreover, *Glut1* seems to be moderately upregulated by BMP treatment, which explains the slight increase in basal glucose uptake (1.3 fold compared to control) observed in BMP-stimulated mature adipocytes.

We propose that GLUT4 is upregulated by BMP/Smad activation, subsequent to PPARγ induction. The master regulator of adipogenesis and adipocyte function, PPARγ, is significantly upregulated as early as 4 h after BMP stimulation on mRNA level and peaks at 8 h on protein level in 3T3-L1 cells. The promoter region of *Pparg* contains six conserved Smad1/5/8 binding sites, further indicating *Pparg* as a direct BMP-Smad target, also in terminally differentiated cells^49^. We clearly show that the BMP-stimulated upregulation of PPARγ is causative for the insulin-sensitizing effect since it is attenuated in PPARγ-depleted cells. This confirms previous studies on decreased GLUT4 levels by reduction of PPARγ^50^ (Supplementary Fig. S3e) and presents BMPs as a promising new insulin-sensitizing stimulus. Interestingly, costimulation with BMPs and Rosiglitazone led to even higher insulin-mediated glucose uptake rates compared to Rosiglitazone alone and further confirms PPARγ as the central mediator. From this we conclude that the PPARγ-mediated upregulation of GLUT4 is crucial for the insulin-sensitizing effect of BMP.

Besides the Smad pathway, BMPs also induce the MAPK pathway. The activation of p38 MAPK by BMP2/6 seen here might explain previous results by others on MAPK modulating the transcriptional activity of PPARγ^27,51^. In future studies it might be interesting to examine whether BMP2/6 heterodimers, which were shown to more potently activate Smad- and non-Smad pathways in hES cells^52^, might also induce stronger downstream effects in mature adipocytes.

To analyze comprehensive transcriptional changes in mature adipocytes stimulated with BMPs, we performed RNA-seq. Besides the classical BMP-target genes as *Smad6, Smad7, Smad9*, and *Id1-4* also PPARγ target genes such as *Lpl, Plin* and *Fasn*, involved in glucose and lipid metabolism and the PPARγ co-receptor Rxra *Rxra XRa* were upregulated. Conversely, *Fatp1* was considerably downregulated after 10 h BMP treatment, while time-resolved analyses revealed that it is upregulated in control conditions. This suggests a compensatory mechanism to overcome the prolonged experimental starvation by upregulating the LCFA transporter and to import more fatty acids. BMP stimulation seems to prevent this effect, indicating that *de novo* lipogenesis might be enhanced, which is reflected by the upregulation of *Fasn*. The adipokine Leptin was reported to not only regulate appetite, energy balance, and adiposity but also to function as a proinflammatory cytokine implicated in tumorigenesis and hypertension^53^. Since in obesity, chronic low-grade inflammation in adipose tissue and high blood pressure accompany insulin-resistance, the BMP-mediated downregulation of proinflammatory factors shown here is of great importance. Both BMP4 and BMP6 have been reported to exert anti-inflammatory effects in human adipocytes and in the context of renal fibrosis in diabetes, respectively^54,55^.

**Figure 6:**
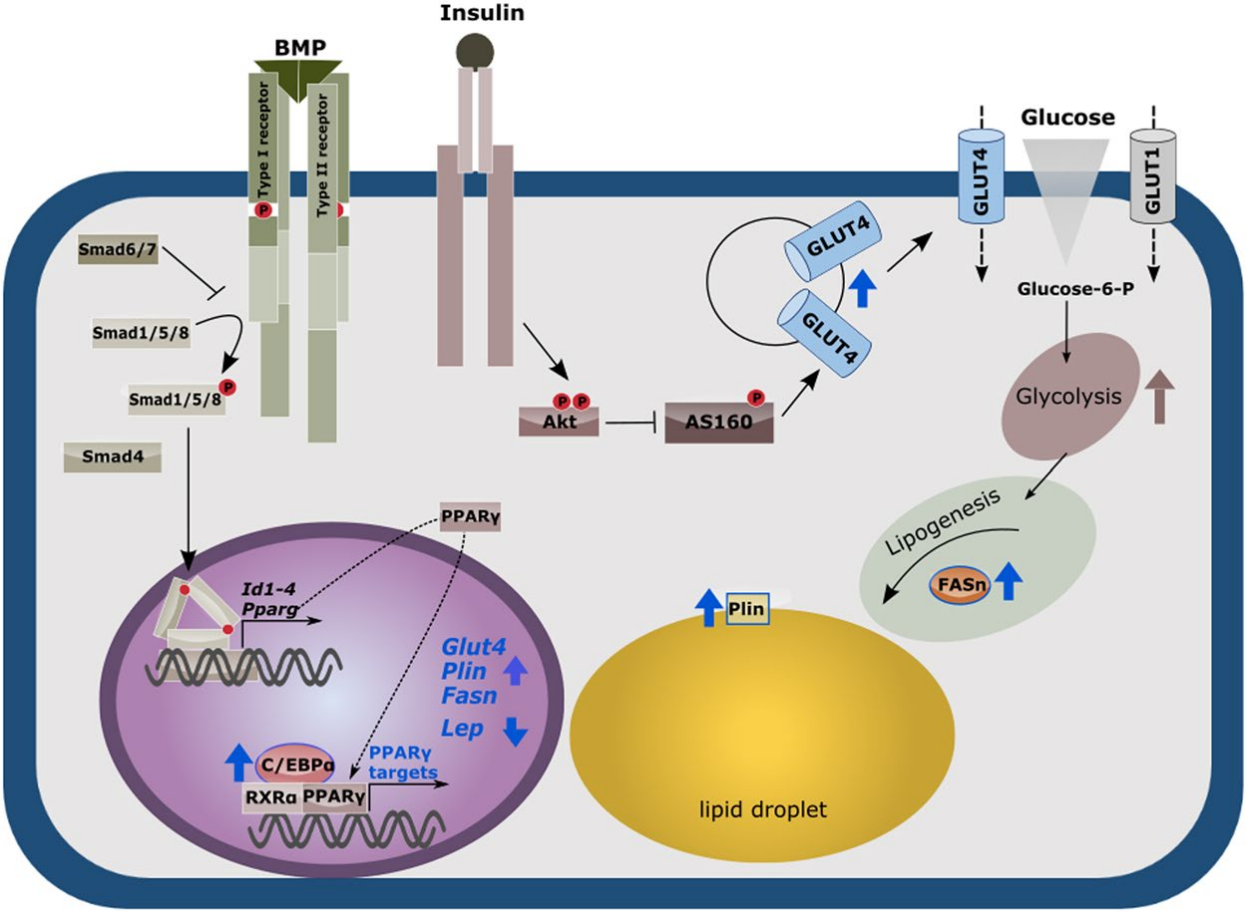
BMP2 or BMP6 signaling enhances adipocyte function. The BMP/Smad pathway leads to transcriptional upregulation of target genes like *Id1-4* and *Pparg*. PPARγ, in turn, acts as a transcription factor on known target genes like *Glut4, Fasn, Plin* and *Lpl* enabling mature adipocytes to more efficiently take up glucose (GLUT4) upon insulin receptor signaling, which results in a higher glycolysis rate and most likely enhanced lipogenesis (Fatty acid synthase) and storage (Perlipin) of lipids.

Based on our findings, we propose BMP2 and BMP6 as new potent insulin-sensitizers in adipocytes. Interestingly, these BMPs upregulate a very specific set of target genes refining the metabolic capacity of mature adipocytes (Fig. 6). Their effect is mediated by the transcriptional upregulation of PPARγ and subsequent modulation of PPRE-containing target genes. This mechanism clearly differs from the action of known insulin-sensitizers like thiazolidinediones acting as nuclear receptor agonists. It is tempting to speculate, that upregulation of these specific BMPs in adipose tissue is a compensatory mechanism of the body to cope with high glucose concentrations and emerging insulin resistance. This is in line with the finding that *BMP2* mRNA is most upregulated in adipose tissue of individuals with impaired glucose tolerance^26^. We propose a dual mechanism: (1) promoting differentiation of residual preadipocytes in the tissue in combination with (2) PPARγ induction, targeting GLUT4 and genes related to lipid metabolism in mature adipocytes, to exert maximal glucose metabolism. Thus, the modulation of BMP signal transduction regulating PPARγ and its target genes in adult tissues harbors a great potential to treat insulin resistance.

## Methods

### Reagents/Cytokines

Recombinant human BMP2 was kindly provided by Walter Sebald (University of Würzburg, Germany) and used at a final concentration of 10 nM or 20 nM. BMP6 was provided by Slobodan Vukicevic (University of Zagreb, Croatia) and used at a final concentration of 10 nM. TGF-β1 was obtained from PeproTech and used at 100 pM. Recombinant insulin (Roche Diagnostics) was used at a final concentration of 100 nM and Rosiglitazone (Sigma-Aldrich) at 10 µM.

### Cell culture and transfections

3T3-L1 preadipocytes were cultured and differentiated in high glucose medium (25 mM) as described previously^56^. Experiments were carried out at d8 or d9 after induction of differentiation. GLUT4myc7-GFP 3T3-L1-preadipocytes were sorted for the intermediate high GFP-expressing population and differentiated like 3T3-L1^36^. Insulin-resistant 3T3-L1 were generated via chronic insulin-stimulation^37^. Knockdown of PPARγ by 75 nmol siRNA oligonucleotides (ON-TARGET plus PPARγ Smart Pool #1 CGAAGAACCAUCCGAUUGA, #2 ACCCAAUGGUUGCUGAUUA, #3 UCACAAUGCCAUCAGGUUU, #4 CGACAUGAAUUCCUUAAUG and ON-TARGET plus non-targeting siRNA #1 UGGUUUACAUGUCGACUAA; Dharmacon/Thermo Fischer) was performed in mature 3T3-L1 cells by electroporation (Neon transfection kit, Thermo Fisher).

### 2-Deoxyglucose uptake

3T3-L1 cells were incubated in FCS- and glucose-free DMEM at 37 °C for 2 h and subsequently stimulated with 100 nM insulin (Roche Diagnostics) or PBS for 30 min. Subsequently, 0.5 µCi/ml 2-deoxy-D-[^3^H]glucose (ARC American Radiolabeled Chemicals Inc.) were added for 10 min at 37 °C. Before lysis in 0.1 M NaOH, cells were washed once with KRH buffer containing 25 mM D-glucose and twice with PBS. Liquid scintillation (Ultima Gold Scintillation Cocktail, Perkin Elmer) measurements were performed in triplicates and counts per minute (CPM) were normalized to total protein determined by BCA protein assay (Thermo Fisher).

### Flow cytometric analyses and immunofluorescence microscopy

3T3-L1 cells were trypsinized, washed with PBS, stained with BODIPY 493/503 (Molecular Probes, 1 µg/ml final) for 5 min, and analyzed via flow cytometry (BD CantoII or Beckman Coulter Epics XL-MCL). GLUT4myc7-GFP-assays were performed as described previously^36^. Intracellular GLUT4 (3G10A3, Thermo Fisher) staining of 3T3-L1 cells was performed after treatment with FACS Lysing Solution and Permeabilizing Solution 2 (BD) according to manufacturer’s instructions. For epifluorescence microscopy (Zeiss Axiovert 200) using AxioVision software, cells were fixed (4% PFA), permeabilized (0.2% Triton X-100), and stained with BODIPY 493/503 (1 µg/µl, Thermo Fisher), DAPI (0.2 µg/µl, Sigma-Aldrich) and Phalloidin (1:200, Santa Cruz). At least 15,000 cells per sample were analyzed.

### Extracellular Flux (XF) measurements

Adipocytes were differentiated until day 7, reseeded in XF96 cell culture microplates (Agilent Technologies) at a density of 15.000/well, and stimulated with BMPs or PBS overnight in medium containing 0.5% FCS. Before measurements, cells (d8) were incubated for 1 h in assay medium (phenol-free DMEM, 1.85 g/L NaCl, 2 mM L-glutamine, 5.5 mM D-glucose, pH 7.4) in a CO_2_ free incubator at 37 °C. ECAR and OCR were measured simultaneously in multiple series of 4 min mixing and 2 min measurement steps with a Seahorse XFe96 Analyzer (Agilent Technologies) as described previously^57^. Final concentrations of 100 nM insulin, 10 mM D-glucose, and 100 nM 2-DG were injected at indicated time points. Values were normalized to total protein content determined by BCA protein assay.

### Immunoblotting

Phosphoprotein assays and Western blot analyses of cell lysates were performed as described previously^58^. Antibodies were used following manufacturer’s recommendations: PPARγ (#2435), P-Smad1/5/8 (#9511 L), P-Smad 1/5/9 (#13820), P-ERK1/2 (#4370), P-AS160 (#4288), AS160 (#2670, GAPDH (#2118), P-Akt_Ser473_ (#4060 L)_,_ P-Akt_Thr308_ (#2965 S) and Akt (pan) (#4691) Smad 1 (#6944) p38 (#8690) antibodies were purchased from Cell Signaling, the anti-active p38 antibody (1:1000) from Promega and the β-Actin antibody (1:5000, A5441) from Sigma-Aldrich.

### Quantitative RT-PCR and whole transcriptome analysis

RNA was purified using NucleoSpin RNA II (Machery-Nagel) according to manufacturer’s instructions. 1 µg RNA was reverse transcribed into cDNA (M-MLV reverse transcriptase, Promega). Quantitative RT-PCR was performed using SYBR Green Master Mix, StepOne Plus and StepOne Software 2.3 (Applied Biosystems). Target gene expression was quantified relative to *18S rRNA* using the ΔΔ_CT_ method including primer efficiency^59^. Measurements were done in technical triplicates. For RNAseq, 1 µg RNA per sample was subjected to library preparation (NEBNext Ultra RNA Library Prep Kit, NEB) and sequenced by the BCRT NGS core facility (HiSeq. 1500 RNA sequencer, Illumina).

### Statistical analyses

Statistical analyses for qRT-PCR, glucose uptake and glycolysis were performed using GraphPad Prism 6 (GraphPad Software Inc.). Normal distribution of the qRT-PCR results was tested with the D’Agostino & Pearson omnibus normality test. In the case of normal distribution, the ANOVA and Bonferroni post-hoc test were used to check for statistical significance. Otherwise the Kruskal-Wallis test and a Dunn’s multiple comparisons test were applied.

Normal distribution of the results for glucose uptake and extracellular flux measurements was tested with the Kolmogorov Smirnov test. In the case of normal distribution, the ANOVA and Bonferroni post-hoc test were used to check for statistical significance. Otherwise the Kruskal-Wallis test and a Dunn’s multiple comparisons test were applied. For all experiments statistical significance was assigned, in case of *p < 0.05 **p < 0.01 and ***p < 0.001.

RNA-seq data was mapped to the mouse genome (mm9). Differential expression was determined by DEseq. 2 analysis. Cutoff values for statistical significance were defined based on volcano plots. Genes with an absolute fold change > 1.5 and adjusted p-value < 0.01 were assessed to be significantly differentially expressed. For heatmaps, RPKM-values of these genes were scaled to a mean of zero and a standard deviation of one. Based on these vectors hierarchical clustering was performed and five clusters were identified by visual inspection. MGSA and Parent-Child-Intersection method of the Ontologizer^60^ were used for GO analyses. GO terms with Bonferroni corrected p < 0.05 were considered as significant. PPARγ target genes were derived from Nackachi *et al*.^29^.

## Electronic supplementary material

Supplementary Information
