# Supplementary material for: BMPs as new insulin sensitizers: enhanced glucose uptake in mature 3T3-L1 adipocytes via PPARγ and GLUT4 upregulation
Source: Sci Rep. 2017 Dec 8;7:17192. doi: 10.1038/s41598-017-17595-5 (PMC5722815; doi:10.1038/s41598-017-17595-5)
Supplement: Supplementary file 1 — Supplementary Information [file 41598_2017_17595_MOESM1_ESM.pdf]

# BMPs as new insulin sensitizers: enhanced glucose uptake in mature 3T3-L1 adipocytes via PPARγ and GLUT4 upregulation

Isabelle Schreiber[1,2], Gina Dörpholz[1], Claus-Eric Ott[3], Bjørt Kragesteen[2,4], Nancy Schanze[5], Cory Thomas Lee[1], Josef Köhrle[5], Stefan Mundlos [2,3,4], Karen Ruschke[1], Petra Knaus*[1,2]

1) Institute of Chemistry and Biochemistry - Biochemistry, Berlin, Germany
2) Berlin-Brandenburg School for Regenerative Therapies (BSRT), Berlin, Germany
3) Institute for Human Genetics and Medical Genetics, Charité - Universitätsmedizin Berlin, Germany
4) Max Planck Institute for Molecular Genetics, Berlin
5) Institute for Experimental Endocrinology, Charité-Universitätsmedizin Berlin, Germany

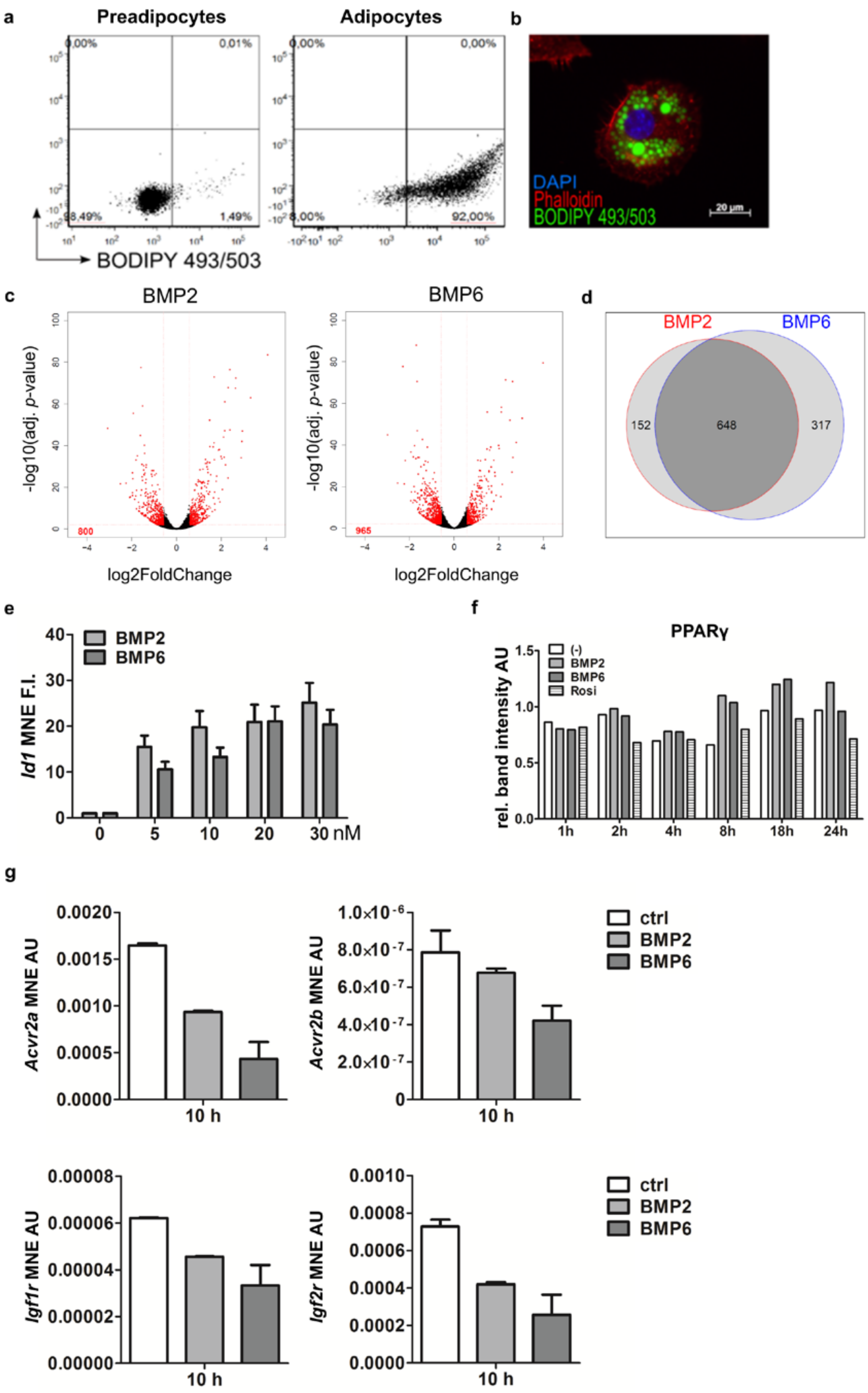

a
Preadipocytes
Adipocytes
BODIPY 493/503
b
DAPI
Phalloidin
BODIPY 493/503
20 µm
c
BMP2
BMP6
-log10(adj. p-value)
log2FoldChange
800
965
d
BMP2
BMP6
152
648
317
e
BMP2
BMP6
Id1 MNE F.I.
0
5
10
20
30 nM
f
PPARγ
(-)
BMP2
BMP6
Rosi
rel. band intensity AU
1h
2h
4h
8h
18h
24h
g
ctrl
BMP2
BMP6
Acvr2a MNE AU
Acvr2b MNE AU
Igf1r MNE AU
Igf2r MNE AU
10 h

Supplementary Figure S1.

(a) Preadipocytes (left) and differentiated 3T3-L1 adipocytes (right) were stained with BODIPY 493/503 eight days after induction of adipogenesis and analyzed by flow cytometry. (b) Adipocytes were treated as in (a) and analyzed by immunofluorescence. (c) Volcano plots showing all significantly regulated genes. Based on the plots genes with ≥ 1.5 fold differential expression by 10 h of BMP2 or BMP6 stimulation were selected (n=2) for heatmap generation and clustering (n=2). (d) Venn diagram showing the overlap of BMP2 and BMP6 regulated genes- the union was used for heatmap generation and further analysis (1084 genes). (e) Adipocytes were treated with the indicated concentrations of BMP2 or BMP6 and analyzed for *Id1* expression via qRT-PCR. Bar chart represents means + SEM of triplicate measurements. (f) Quantification of band intensities normalized to β-Actin of the blot shown in Fig. 1a (g) Adipocytes were treated as outlined in Fig. 2. Following 10 h stimulation with BMP2 or BMP6 gene expression of Acvr2a, Acvr2b, Igf1r and Igf2r were downregulated. Data are presented as means + SEM of two independent experiments with triplicate measurements different from samples used in the RNA-Seq experiment. All genes were selected from Cluster D.

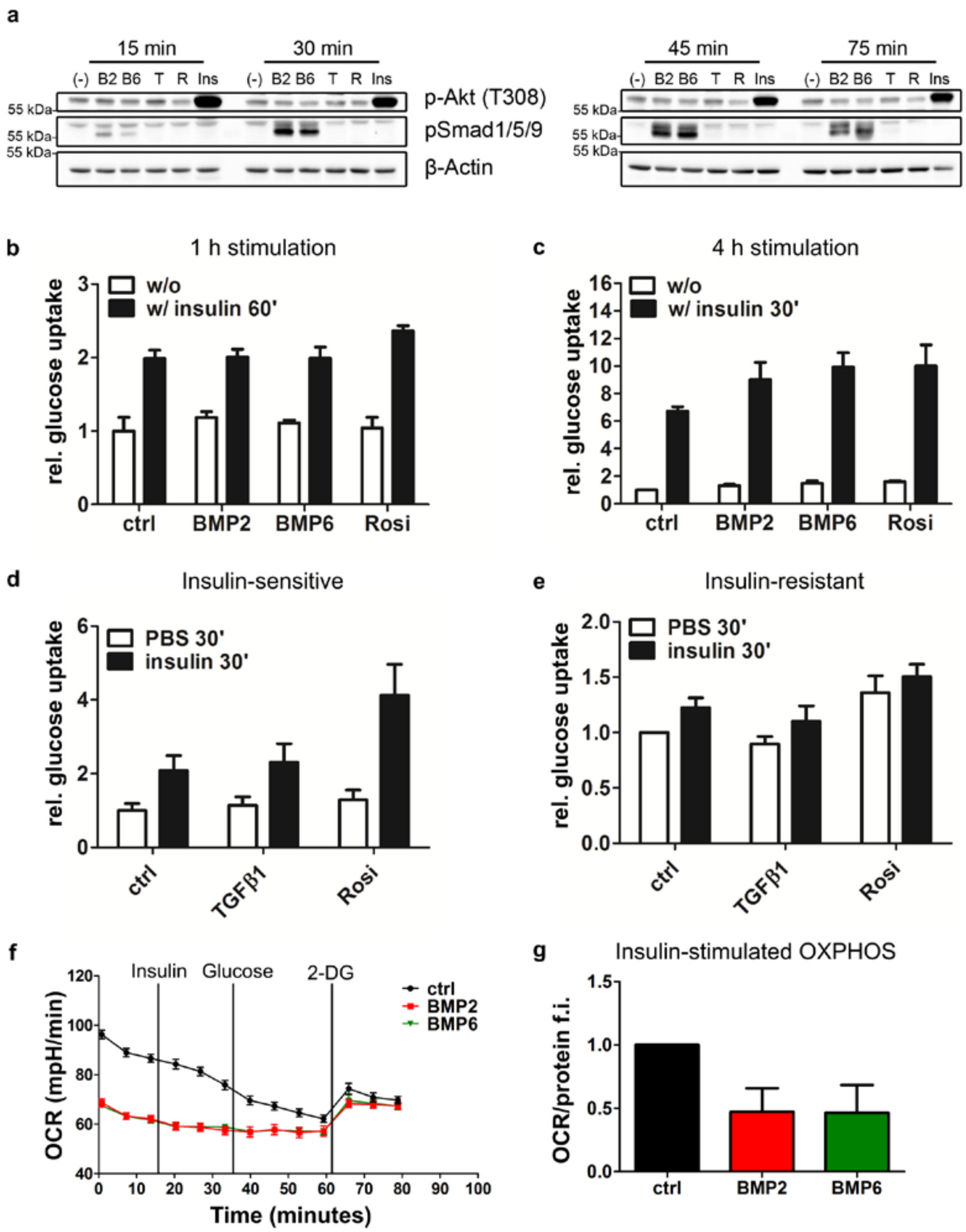


Supplementary Figure S2.

(a) Mature adipocytes (d8) were stimulated with PBS (-), 10 nM BMP2 (B2), 10 nM BMP6 (B6), 100 pM TGFβ1 (T), 10 µM Rosiglitazone (R) or 100 nM insulin (I) for indicated time points. Phosphorylation of Smad1/5/9 and Akt at Thr308 was analyzed via immunoblotting using specific antibodies. (b) Adipocytes were stimulated with PBS, BMP2, BMP6 or Rosi and simultaneously with PBS (w/o) or insulin (w/insulin) as indicated for 60 minutes. 2-deoxy-D[$^3$H] glucose uptake was measured and normalized to total protein content. Bar chart depicts means + SEM of triplicate measurements. (c) Adipocytes were treated with PBS, BMP2, BMP6 or Rosiglitazone for 4 h before 2-deoxy-D[$^3$H] glucose uptake ± insulin for 30 minutes. Bar chart depicts means + SEM of triplicate measurements. (d) Mature adipocytes or (e) insulin-resistant adipocytes were stimulated with PBS, 100 pM TGF-β1 or Rosiglitazone for 18 h. 2-deoxy-D[$^3$H] glucose uptake of adipocytes was measured after stimulation with PBS (white bars) or 100 nM insulin (black bars) for 30 minutes and normalized to total protein content. Bar charts depict means + SD, representative experiment for (d); means + SEM n=3 for (e). (f) Respective OCR measurement to the

experiment in Fig. 5 c. (g) Quantification of insulin-stimulated OXPHOS (measurement 6 (36 minutes) minus measurement 3 (18 minutes) Data are means + SEM, n≥3.

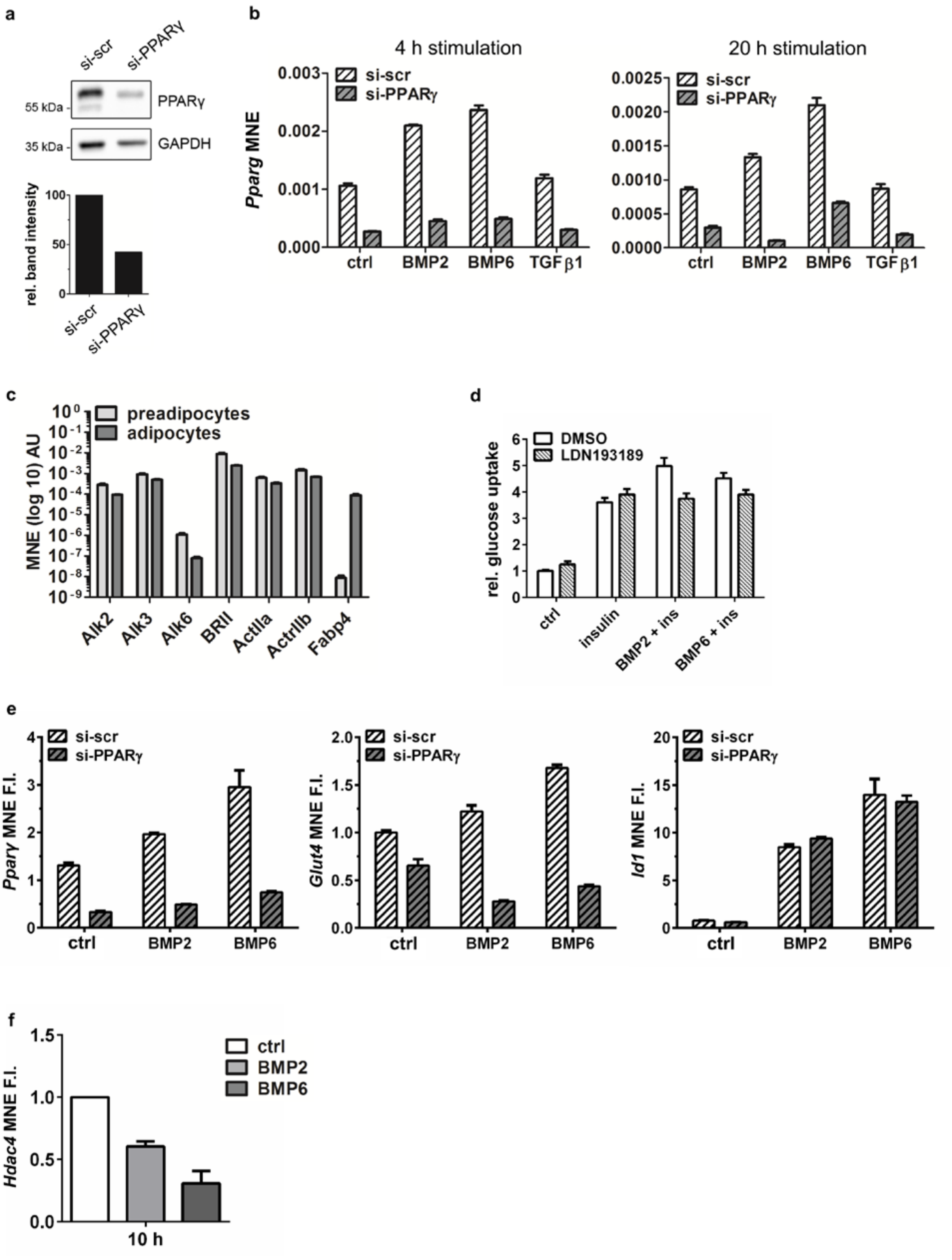

Supplementary Figure S3.

3T3-L1 adipocytes were electroporated with non-targeting scrambled siRNA (si-scr) or siRNA targeting PPARγ (si-PPARγ) and incubated for 24 h before stimulation with PBS, BMP2, BMP6 or TGF-β1 for 24 h (a) or 4 h and 20 h (b). (a) PPARγ knockdown efficiency on protein level was assessed via immunoblotting. Quantification of total PPARγ protein levels normalized to GAPDH reveals a 60% knockdown efficiency. (b) RNA was isolated, reverse-transcribed and subjected to gene expression analysis via qRT-PCR for *Pparγ* mRNA. Data are mean normalized expression (MNE) + SEM from triplicate measurements. (c) BMP receptor expression levels of 3T3-L1 preadipocytes (light gray bars) vs. mature adipocytes (dark gray bars) were determined by qRT-PCR. (d) Mature adipocytes (d8) were treated with 5 μM LDN193189 or DMSO for 30 min previous to stimulation with PBS, BMP2, or BMP6 for 18 h. Insulin-stimulated (30 minutes) 2-deoxy-D[3H] glucose uptake was measured. The bar chart depicts means + SEM from triplicate measurements normalized to total protein content. (e) 3T3-L1 adipocytes were transfected with scrambled siRNA (si-scr) or siRNA targeting PPARγ (si-PPARγ) and incubated for 24 h before stimulation with BMPs, insulin or Rosiglitazone for 20 h. Samples were analyzed for mRNA expression of *Pparγ*, *Glut4* and *Id1* in triplicate measurements. (f) Following 10 h stimulation with BMP2 or BMP6 gene expression of *Hdac4* was downregulated. Adipocytes were treated as outlined in Fig. 2.Data are presented as means + SEM of two independent experiments different from samples used in the RNA-Seq experiment. *Hdac4* was selected from Cluster E.

Supplementary Table 1.

RNAseq analysis confirms *in silico*-predicted and known PPARγ target genes. The data set presented in Figure 2 was compared with the *in silico*, ChIP-on-chip and microarray expression data from Nakachi *et al.* [1]. Known PPARγ target genes are indicated with §, predicted PPARγ target genes, harboring PPRE consensus sites in or in close proximity to their promoters are indicated with #. Asterisks denote PPARγ target genes selected for qRT-PCR validation. The gene highlighted in light gray was differentially expressed only in BMP2-treated samples, whereas genes marked in dark gray were significantly regulated only in BMP6-treated samples, respectively. All other genes were significantly regulated in both stimulation conditions.

| Gene.Symbol | Ensembl.Gene.ID | Gene.Description.Ensembl | PPRE.Match | BMP2.FC | BMP2.padj | BMP6.FC | BMP6.padj |
|---|---|---|---|---|---|---|---|
| Plch2 | ENSMUSG00000029055 | phospholipase C-like 4 | # | 0.546 | 5.63E-03 | 0.632 | 3.68E-02 |
| Bmf | ENSMUSG00000040093 | Bcl2 modifying factor | # | 0.632 | 1.83E-03 | 0.519 | 1.49E-06 |
| Cpne3 | ENSMUSG00000028228 | copine III | # | 0.702 | 1.71E-04 | 0.626 | 1.61E-07 |
| Cxcl1 | ENSMUSG00000029380 | chemokine (C-X-C motif) ligand 1 | # | 0.464 | 2.67E-04 | 0.503 | 1.01E-03 |
| Cycs | ENSMUSG00000058927 | cytochrome c, somatic | # | 1.387 | 1.22E-04 | 1.604 | 2.90E-09 |
| Ephb3 | ENSMUSG00000005958 | Eph receptor B3 | # | 1.568 | 2.41E-08 | 1.299 | 2.58E-03 |
| Gata3 | ENSMUSG00000015619 | GATA binding protein 3 | # | 2.051 | 5.40E-06 | 1.888 | 7.49E-05 |
| Ggta1 | ENSMUSG00000035778 | glycoprotein galactosyltransferase alpha 1, 3 | # | 0.482 | 3.28E-04 | 0.472 | 1.50E-04 |
| Gjc1 | ENSMUSG00000047197 | gap junction membrane channel protein chi 1 | # | 0.665 | 1.55E-07 | 0.660 | 8.83E-08 |
| Gnb5 | ENSMUSG00000032192 | guanine nucleotide binding protein, beta 5 | # | 1.353 | 4.98E-03 | 1.529 | 1.64E-05 |
| Osbpl5 | ENSMUSG00000037606 | oxysterol binding protein-like 5 | # | 0.713 | 1.57E-04 | 0.626 | 2.99E-08 |
| Paqr4 | ENSMUSG00000023909 | progestin and adipoQ receptor family member IV | # | 2.398 | 3.10E-21 | 2.352 | 3.21E-20 |
| Rufy3 | ENSMUSG00000029291 | RUN and FYVE domain containing 3 | # | 2.643 | 1.48E-23 | 2.705 | 1.75E-24 |
| Sertad1 | ENSMUSG00000008384 | SERTA domain containing 1 | # | 2.015 | 1.51E-16 | 2.318 | 1.08E-23 |
| Synpo | ENSMUSG00000043079 | synaptopodin | # | 0.620 | 1.57E-03 | 0.575 | 1.27E-04 |
| Tgif1 | ENSMUSG00000047407 | TG interacting factor | # | 0.534 | 3.92E-05 | 0.520 | 1.38E-05 |
| Xpr1 | ENSMUSG00000026469 | xenotropic and polytropic retrovirus receptor 1 | # | 0.649 | 1.55E-07 | 0.562 | 4.66E-13 |
| Snai1 | ENSMUSG00000042821 | snail homolog 1 (Drosophila) | § | 2.232 | 1.16E-22 | 1.923 | 5.18E-15 |
| Sprr1a | ENSMUSG00000050359 | small proline-rich protein 1A | § | 0.290 | 2.14E-10 | 0.353 | 1.49E-07 |
| Vegfa | ENSMUSG00000023951 | vascular endothelial growth factor A | § | 1.578 | 1.06E-05 | 1.521 | 5.19E-05 |
| Abcg2 | ENSMUSG00000029802 | ATP-binding cassette, sub-family G (WHITE), member 2 | § | 2.183 | 4.62E-16 | 2.195 | 2.77E-16 |
| Acsl1 | ENSMUSG00000018796 | acyl-CoA synthetase long-chain family member 1 | § | 1.878 | 2.35E-03 | 1.728 | 7.63E-03 |
| Aqp7 | ENSMUSG00000028427 | aquaporin 7 | § | 2.107 | 3.27E-10 | 1.991 | 8.74E-09 |
| Ccnd1 | ENSMUSG00000070348 | cyclin D1 | § | 0.580 | 3.37E-05 | 0.566 | 1.11E-05 |
| Cdkn1a | ENSMUSG00000023067 | cyclin-dependent kinase inhibitor 1A (P21) | § | 0.642 | 1.25E-08 | 0.647 | 2.34E-08 |
| Cebpa | ENSMUSG00000034957 | CCAAT/enhancer binding protein (C/EBP), alpha | § | 1.572 | 1.67E-04 | 1.521 | 4.41E-04 |
| Cebpb | ENSMUSG00000056501 | CCAAT/enhancer binding protein (C/EBP), beta | § | 1.462 | 8.01E-07 | 1.773 | 4.45E-15 |
| Cidec | ENSMUSG00000030278 | cell death-inducing DFFA-like effector c | § | 2.355 | 1.08E-14 | 2.379 | 4.49E-15 |
| Fabp5 | ENSMUSG00000027533 | fatty acid binding protein 5, epidermal | § | 1.593 | 6.57E-06 | 1.778 | 7.20E-09 |
| *Fasn | ENSMUSG00000025153 | fatty acid synthase | § | 1.991 | 2.96E-11 | 1.528 | 1.16E-04 |
| Gpd1 | ENSMUSG00000023019 | glycerol-3-phosphate dehydrogenase 1 (soluble) | § | 2.526 | 1.51E-20 | 2.385 | 4.29E-18 |
| Hgf | ENSMUSG00000028864 | hepatocyte growth factor | § | 2.263 | 3.91E-08 | 1.875 | 4.65E-05 |
| Lamb3 | ENSMUSG00000026639 | laminin, beta 3 | § | 1.817 | 5.13E-05 | 1.766 | 1.06E-04 |
| *Lep | ENSMUSG00000059201 | leptin | § | 0.585 | 2.24E-02 | 0.530 | 2.95E-03 |
| *Lpl | ENSMUSG00000015568 | lipoprotein lipase | § | 1.642 | 8.11E-12 | 1.548 | 2.61E-09 |
| Nr4a1 | ENSMUSG00000023034 | nuclear receptor subfamily 4, group A, member 1 | § | 0.453 | 3.31E-09 | 0.444 | 1.13E-09 |
| Pck1 | ENSMUSG00000027513 | phosphoenolpyruvate carboxykinase 1, cytosolic | § | 1.744 | 2.23E-12 | 1.667 | 1.59E-10 |
| *Plin | ENSMUSG00000030546 | perilipin | § | 1.482 | 4.79E-03 | 1.517 | 1.73E-03 |
| *Slc27a1 | ENSMUSG00000031808 | solute carrier family 27 (fatty acid transporter), member 1 | § | 0.533 | 2.85E-27 | 0.561 | 6.10E-23 |
| Cdh23 | ENSMUSG00000012819 | cadherin 23 (otocadherin) | # | 0.685 | 3.73E-02 | 0.606 | 2.14E-03 |
| Chd3 | ENSMUSG00000051605 | chromodomain helicase DNA binding protein 3 | # | 0.780 | 1.06E-01 | 0.644 | 4.18E-04 |
| Vps13d | ENSMUSG00000073719 | vacuolar protein sorting 13 D (yeast) | # | 0.799 | 2.26E-01 | 0.600 | 2.26E-04 |
| Abca1 | ENSMUSG00000015243 | ATP-binding cassette, sub-family A (ABC1), member 1 | § | 0.837 | 3.27E-01 | 0.665 | 1.44E-03 |
| Fabp4 | ENSMUSG00000062515 | fatty acid binding protein 4, adipocyte | § | 1.129 | 2.86E-01 | 1.503 | 1.51E-07 |

References

1 Nakachi, Y. *et al.* Identification of novel PPARgamma target genes by integrated analysis of ChIP-on-chip and microarray expression data during adipocyte differentiation. *Biochem Biophys Res Commun* **372**, 362-366, doi:10.1016/j.bbrc.2008.05.037 (2008).
